# Investigation of Multi-Subunit *Mycobacterium tuberculosis* DNA-Directed RNA Polymerase and Its Rifampicin Resistant Mutants

**DOI:** 10.3390/ijms24043313

**Published:** 2023-02-07

**Authors:** Mokgerwa Zacharia Monama, Fisayo Olotu, Özlem Tastan Bishop

**Affiliations:** Research Unit in Bioinformatics (RUBi), Department of Biochemistry and Microbiology, Rhodes University, Makhanda 6139, South Africa

**Keywords:** antitubercular drug resistance, missense mutations, *Mycobacterium tuberculosis*, rifampicin, RNA polymerase, RNAP processivity

## Abstract

Emerging *Mycobacterium tuberculosis* (*Mtb*) resistant strains have continued to limit the efficacies of existing antitubercular therapies. More specifically, mutations in the RNA replicative machinery of *Mtb*, RNA polymerase (RNAP), have been widely linked to rifampicin (RIF) resistance, which has led to therapeutic failures in many clinical cases. Moreover, elusive details on the underlying mechanisms of RIF-resistance caused by *Mtb*-RNAP mutations have hampered the development of new and efficient drugs that are able to overcome this challenge. Therefore, in this study we attempt to resolve the molecular and structural events associated with RIF-resistance in nine clinically reported missense *Mtb* RNAP mutations. Our study, for the first time, investigated the multi-subunit *Mtb* RNAP complex and findings revealed that the mutations commonly disrupted structural–dynamical attributes that may be essential for the protein’s catalytic functions, particularly at the βfork loop 2, β’zinc-binding domain, the β’ trigger loop and β’jaw, which in line with previous experimental reports, are essential for RNAP processivity. Complementarily, the mutations considerably perturbed the RIF-BP, which led to alterations in the active orientation of RIF needed to obstruct RNA extension. Consequentially, essential interactions with RIF were lost due to the mutation-induced repositioning with corresponding reductions in the binding affinity of the drug observed in majority of the mutants. We believe these findings will significantly aid future efforts in the discovery of new treatment options with the potential to overcome antitubercular resistance.

## 1. Introduction

Tuberculosis (TB) remains a global threat and has accounted for many global incidences of mortality [1]. Over many decades, various efforts have been directed towards developing effective therapies that could curtail the spread and have led to the discovery of drugs such as isoniazid, rifabutin, pyrazinamide and rifampicin (RIF) [2,3], which have different action mechanisms. RIF is a first-line drug widely used in anti-tubercular therapy, and its high effectiveness is based on broad-spectrum bactericidal activities [2,4,5]. It is a cyclic, semi-synthetic, antimicrobial agent derived from rifamycins [6] and active against slow-growing and non-replicating bacteria, an attribute that underlies the virulence of *Mycobacterium tuberculosis* (*Mtb*), the known TB causative agent [5,7,8,9].

**Figure 1:**
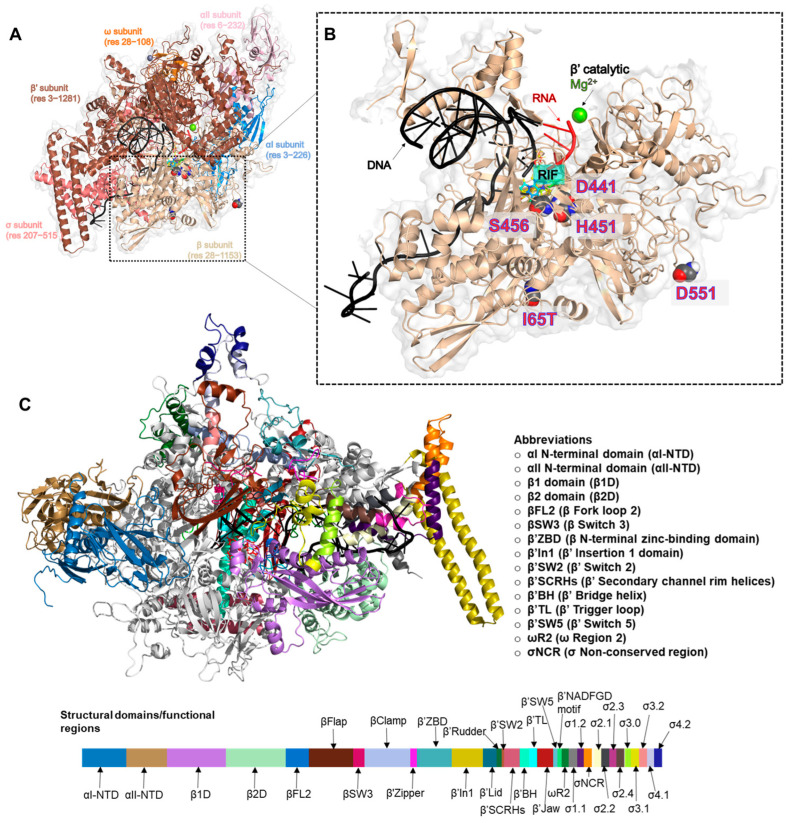
Structural representation of multi-subunit *Mtb*-RNAP with annotated mutation sites and functional regions. (**A**) RNAP is represented as a cartoon and colored blue, light pink, wheat, brown, orange and salmon for the αI, αII, β, β’, ω, and σ subunits, respectively. (**B**) The β subunit is zoomed in to highlight the mutation sites of interest, RIF binding site, β’ASP-coordinated catalytic Mg^2+^ and the bound DNA and RNA. (**C**) 3D structure of *Mtb*-RNAP with mapped functional regions. The annotated color bar represents the corresponding regions on the protein.

Mechanistically, RIF binds specifically to the bacterial DNA-directed RNA polymerase (RNAP), a protein that has been widely studied for its essential roles in transcriptional regulation [10,11,12]. Structurally, the active multi-subunit RNAP core is conserved across all domains of life with some discrepancies due to lineage-specific domain insertions [13,14,15,16]. The enzymatic core is essentially comprised of the αI and αII homodimer, β, β’ and ω, which are encoded by rpoA, rpoB, rpoC and rpoZ genes, respectively [17] (Figure 1A). Functionally, the αI and αII N-terminal domains (NTDs) play roles in transcriptional regulation and RNAP assembly [18], while the ω subunit additionally plays roles in, but not limited to, the folding and physical protection of the β’ [19,20]. The β and β’ subunits each make up a pincer of the enzyme’s characteristic crab claw shape and form the primary channel through which DNA can reach the active site for the elongation of nascent RNA [18,21] (Figure 1B). Completion of the holoenzyme assembly is seen with the association of the σ factor, which is necessary for promoter-DNA recognition and unwinding [22,23,24]. Collectively, these subunits and their composite (sub)domains (Figure 1C) have been widely characterized for their structure–function relationships in many structural studies over the last decade [16,17].

To achieve its anti-tubercular activity, RIF targets the β subunit of *Mtb*-RNAP, thus preventing the elongation of 2–3 nucleotide-long RNA transcripts through steric occlusion, effectively trapping the state of abortive transcription [10,11]. The therapeutic efficacy of RIF has, however, been limited by the emergence and spread of RIF-resistant (RIF^R^) *Mtb* strains with reported missense mutations at the RIF binding region, commonly known as the RIF-resistance-determining region (RRDR), negatively impacting on inhibitor specificity and affinity [9,25].

S456L is the most commonly reported *Mtb*-RNAP mutation and is located in cluster I of the RRDR (residues 432–458), proximal to the β fork loop 2 (β FL2) region (residues 459–473) of the protein, and initial studies revealed that it may have impacted on RIF activity via disordering of βFL2 while displaying less fitness cost overall [26]. This was further kinetically expounded by Stefan et al. [27] where the *Mtb*-RNAP enzymatic activity of S456L and the other most commonly observed cluster I mutations (D441V and H451Y) were also delineated. The clinical effects of H451D, H451L, H451N and H451R were also reported through several studies encompassing multiple populations worldwide [28]. Several in silico studies have attempted to investigate the binding of RIF at the *Mtb*-RNAP β subunit, thus providing mechanistic insights into the delimiting effects of these mutations [29,30,31,32]. Some specifically revealed that D441V, H451D, H451R and H451Y mutations may affect RIF activity by altering the critical binding pocket interactions [29,30,31,32]. Moreover, although numerous RRDR and non-RRDR or distal *Mtb*-RNAP mutations have been reported, the majority of clinical cases of RIF inefficacy have been linked to high-confidence mutations mostly occurring at residue positions D441, H451 and S456 located within RRDR cluster I [28]. 

Complementarily, this current study focused on the mostly occurring and high-confidence mutations (D441V, H451D, H451L, H451N, H451R, H451Y, S456L [33] and D551E) [34] (Figure 1B) and investigated their mechanistic effects on the structure and dynamics of the multi-subunit protein as well as on the inhibitory activities of RIF. Also studied is the recently and clinically reported I65T [35], which, similar to D551E, is distally located from the RRDR. Hence, for the first time, we elucidated the underlying mechanisms of RIF-resistance as mediated by these selected missense mutations using the *Mtb*-RNAP multi-subunit that consists of the αI and αII N-terminal domains (αI-NTD & αII-NTD), β and β’ subunits, ω and σ subunits; DNA, RNA and RIF molecules; and Mg^2+^ and Zn^2+^ ions. Our findings revealed that the mutations uniformly disrupted important structural attributes in the protein, particularly βfork loop 2 (βFL2), β’zinc-binding domain (β’ZBD), the β’ trigger loop (β’TL) and β’jaw, which are essential for catalytic RNA processivity. These were in addition to induced alterations of the RIF-binding pocket (RIF-BP), which in turn disoriented the native pose of RIF most favorable for its ability to halt RNA elongation with consequential reductions in binding affinity. We, therefore, believe that these findings will contribute immensely to the structure-based development of new drugs that are able to overcome these mutation-related therapeutic setbacks.

## 2. Results and Discussion

### 2.1. Consequences of High-Incidence Mutations on Structural–Functional Stability

A total of nine clinically relevant missense mutations (I65T, D441V, H451D, H451L, H451N, H451R, H451Y, S456L and D551E) were retrieved from the literature [33,34,35] in line with our intent to detail their mechanistic impacts on *Mtb*-RNAP’s structure and function as well as RIF inhibitory activity. High-quality structural models were obtained for the *wt* and *mt* systems in the RIF-bound forms, which further validates the modelling approach employed [36]. Primarily, the impacts of the mutations relative to the *wt Mtb*-RNAP were examined using the Cα-root mean square deviation (Cα-RMSD), radius of gyration (Rg) and Cα-root mean square fluctuation (Cα-RMSF) metrics. Comparative essential dynamics (ED) was also used to investigate changes in the global structural motions of the protein as induced by the respective *mt*s relative to the *wt* [37]. 

**Figure 2:**
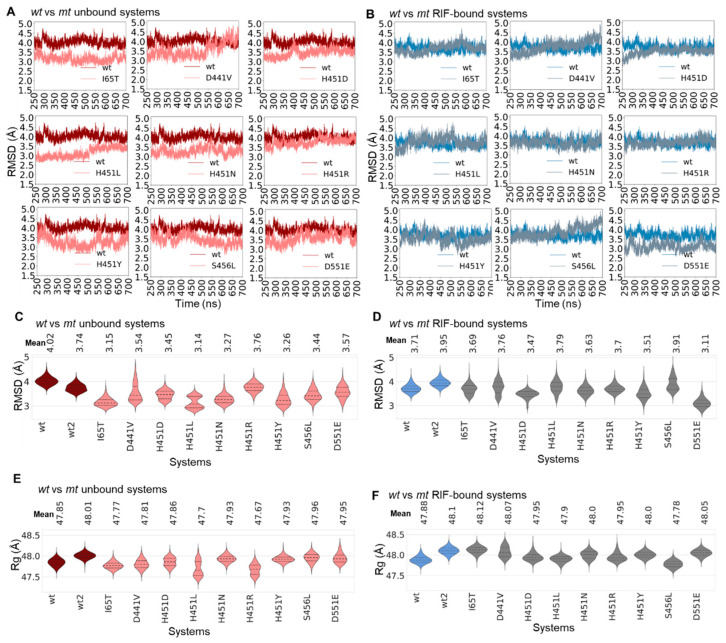
Structural analysis of *Mtb*-RNAP dynamics due to mutation and/or RIF binding. The line plots represent the captured Cα-RMSDs (with reference to their initial structures) for (**A**) RIF-unbound and (**B**) bound *wt* (unbound, maroon; RIF-bound, blue) and *mt* (unbound, orange; RIF-bound, pink) *Mtb*-RNAP proteins. Cα-RMSD distribution violin plots are shown for the (**C**) unbound and (**D**) RIF-bound *wt*s and *mt*s. The mean Cα-RMSD values are indicated above the plots, while the dotted lines on the violins represent the 25th, 50th and 75th quartiles. Similarly ordered Rg distribution violins are also shown for the (**E**) unbound and (**F**) RIF-bound systems. The same coloring scheme used in the line plots is used in the violins.

As shown from the 700 ns RMSD line plots and all versus all RMSD heatmaps, the standard (*wt*) systems were equilibrated around 250 ns (Supplementary Figure S1A–D). Hence, to minimize the noise, the equilibrated part of each of the trajectories (250–700 ns) was employed for all the analyses in this study unless otherwise stated. Cα-RMSD results for the duplicate molecular dynamic (MD) simulation runs showed average differences of 0.28 Å and 0.24 Å, respectively, for the RIF unbound and bound *wt* proteins (Supplementary Figure S1E,F). In the duplicate (control) run, consistent results were observed in the conformational dynamics of the *wt* proteins (bound and unbound) compared to the first, which altogether gave higher confidence that differences observed in the *mt* systems (across the equilibrated timeframes) were most likely caused by the mutations (Figure 2A,B and Figure S2A,B). These altogether provided important insights into the degree to which the mutations altered the conformational stability and motions of *Mtb*-RNAP. Even though we were unable to obtain duplicate MD runs for the mutant systems due to the huge computational cost (>3000 residues), we performed comparative RMSD analyses with each of the *wt* runs to ensure that they did not highly deviate from the control as seen in Figure 2A,B and Figure S2A,B.

Variations in the dynamics of the proteins as possibly induced by mutational or ligand-binding effects were further delineated by the line and violin Cα-RMSD and Rg plots [38,39] with violin peaks showing the highest sampled equilibrium conformation per system and the width indicating the extent of the sampled conformations per equilibria. As shown in Figure 2A, we observed that in the RIF-unbound states, the mutants showed lower RMSDs compared to the wild type. In a similar trend, this event was largely consistent in the second control run (Supplementary Figure S2A). Additionally, from the violin plot (RIF-unbound, Figure 2C), it was observed that the *wt* system, considering both runs, exhibited a more stable distribution, while the majority of the mutants sampled multiple conformations, particularly H451L, D441V, D551E and H451D, which could imply compensatory motions in an attempt to retain structural integrity. More so, while the binding of RIF seemed to further stabilize the dynamics of the *wt* protein consistently for both runs, most of the mutants showed considerable changes in conformation over time in response to RIF binding with relatively lower RMSDs (Figure 2B and Figure S2B). These erratic changes were particularly evident in D551E, H451Y, S456L, I65T and D441V. Comparative violin distribution plots for the RIF-bound systems further detailed the variations in the conformational sampling of the RIF-bound proteins relative to both *wt* runs with multimodal distributions observed particularly in S456L, H451L, D441V, I65T, H451Y and H451D (Figure 2D). These deductions could possibly be further supported by the average RMSD calculations, which showed reduced values for majority of the mutant proteins in the unbound states when compared with estimates for both *wt* runs (Figure 2C). Furthermore, aside from D551E and H451D, which showed considerable reductions in average RMSDs among the RIF-bound *mt* systems, differences were minimal for the other *mt*s when compared to RMSD estimates from both the first and second control runs (Figure 2D). Notwithstanding, the majority of the bound *mt*s exhibited characteristic increases in conformational sampling compared to the *wt* runs that could still indicate the extent to which they were perturbed. Presumably, the stabilizing effect of RIF as seen in the dynamics of the *wt Mtb*-RNAP over time in both runs could indicate a possible inhibitory pattern as previously reported [11], while, on the other hand, decreases in RMSDs as well as structural perturbations observed in most of the *mt*s could corroborate experimental reports on mutant-induced RNAP inefficiencies [27]. 

Rg estimations further revealed that the effects of the mutations on the structural compaction of the protein are majorly inconsequential as seen in almost all the *mt*s with stable Rg distributions comparable to both *wt* runs all in the RIF-unbound state. However, H451R and H451L mutations seem to distinctly impact on the compaction as both *mt*s exhibited multimodal distributions with greater sampling (Figure 2E). More so, there were no notable changes in compactness in the presence of RIF as evident in the two *wt* runs and also for the *mt*s (Figure 2F). Complementarily, differences in average Rg values were minimal across the RIF-bound and unbound systems. 

Comparative ED calculations further revealed different phase space occupancies, mainly across PC2 (captured variance of 10.93% and 12.15%, respectively), while displaying more similarities along PC1 (captured variance of 14.07% and 14.44%, respectively) for the RIF-bound and unbound *wt* systems, as also replicated in the second control run (Supplementary Figure S3). This tool addresses a potential problem of a general ED approach that may occur when comparing separate MD simulations: generation of distinct sets of eigenvector/eigenvalue pairs. Hence, the tool aligns the trajectories first. This might cause a lower PC1 and PC2 values yet yield a common set of eigenvector–eigenvalue pairs that explain the total variance. 

Furthermore, the differences in conformational distributions observed between the two *wt* systems in each state is likely attributed to the discrepancies in conformational sampling and residue fluctuations of some of the subunits as seen in Supplementary Figures S4 and S5, thus resulting in differences in the overall conformations. Regardless, comparatively larger deviations in phase space occupancy along either PC coupled with varying conformational distributions were observed for most of the *mt*s in agreement with the RMSD violin plots. More specifically in the unbound systems, H451D, H451L and H451R showed the largest deviations in occupancies along PC1 relative to both *wt* runs, while occupancy deviations were most notable in H451N, H451R, D551E and D441V along PC2 (Supplementary Figure S3A). In the presence of RIF, all the *mt*s (to a lesser extent, D551E) displayed clear differences along PC1 with H451Y in particular indicating a larger conformational distribution (Supplementary Figure S3B).

Additionally, varying degrees of perturbations occurred across the protein subunits as disparately induced by the effects of the mutations or RIF. These were quantified by the subunit RMSD calculations, which measured the relative stabilities of each *Mtb*-RNAP subunits αI-NTD, αII-NTD, β, β’, ω and σ subunits and could correlate with the extent to which the constituent residues are destabilized as caused by the mutations. As shown in the RMSD data plots (Supplementary Figures S4 and S5), structural occurrences across these subunits were mostly the same for the two *wt* runs in the RIF-unbound and bound forms. Stable distributions were observed for the unbound *wt* runs in the αI-NTD, β and β’ subunits, which constitutes the RIF-BP and the DNA/RNA binding regions. Slight changes in RMSD distributions occurred at these regions in the presence of RIF besides the terminal αI, which exhibited much higher deviations. H451R, H451D, S456L and H451Y, however, showed the largest deviations from the *wt* runs in the β subunit (unbound), while deviations at the largest catalytic subunit, the β’ subunit, were notable in most of the *mt*s compared to the *wt* runs and were most prominent in D441V and H451L. Similarly in the RIF-bound systems, I65T and H541D (multimodal) deviated largely in the β subunit as compared to the *wt* runs, while S456L and H451L also had notable multimodal distributions that could imply mutation-induced changes to the integrity of the region. Notable conformational variations were also observed in the ω subunit in most of the RIF-bound *mt*s relative to the *wt* runs as characterized by multimodal distributions particularly D441V and H451Y. Although minor changes in RMSD distributions were observed for the unbound *mt* αI-NTDs, D441V in particular registered the largest RMSD among all the *mt*s highlighting a unique RIF-binding effect when compared to the two *wt* runs. Lastly, for the DNA-binding σ subunit, while conformational sampling was similar for the *mt*s and *wt*s in the RIF-unbound state, the RIF-bound D441V, S456L, D551E, H451D, H451Y and H451L systems displayed large differences in RMSD and conformational distributions relative to the *wt* runs. Collectively, these disruptions could possibly impact on the catalytic process of the protein to which these subunits are essential. Furthermore, considering the consistencies observed for the first and second control *wt* runs, we proceeded with the initial run for subsequent structural–dynamics analyses of the functional domains RIF-BP, RIF as well as the catalytic nucleic acids.

### 2.2. Mutation-Induced Destabilization of Key Functional Mtb-RNAP Domains

Processive RNA synthesis through addition of nucleoside triphosphates (NTPs) describes the core of *Mtb*-RNAP’s catalytic activities, which is majorly facilitated by several critically important functional domains [21]. More so, mutational effects as well as RIF binding have been shown to alter catalysis by interfering with the nucleotide addition process [10,11,27]. We further investigate herein the complementary effects of these events on functional domain dynamics, details which are elusive in the literature. Accordingly, we employed Cα-RMSD and Cα-RMSF analyses to explore varying instabilities that occurred at specific (sub)domains particularly involved in enzyme catalysis. These include the βFL2 (residues 459–473), β’TL, β’jaw (residues 1039–1218) and the β’ZBD (residues 24–98) (Figure 1C).

**Figure 3:**
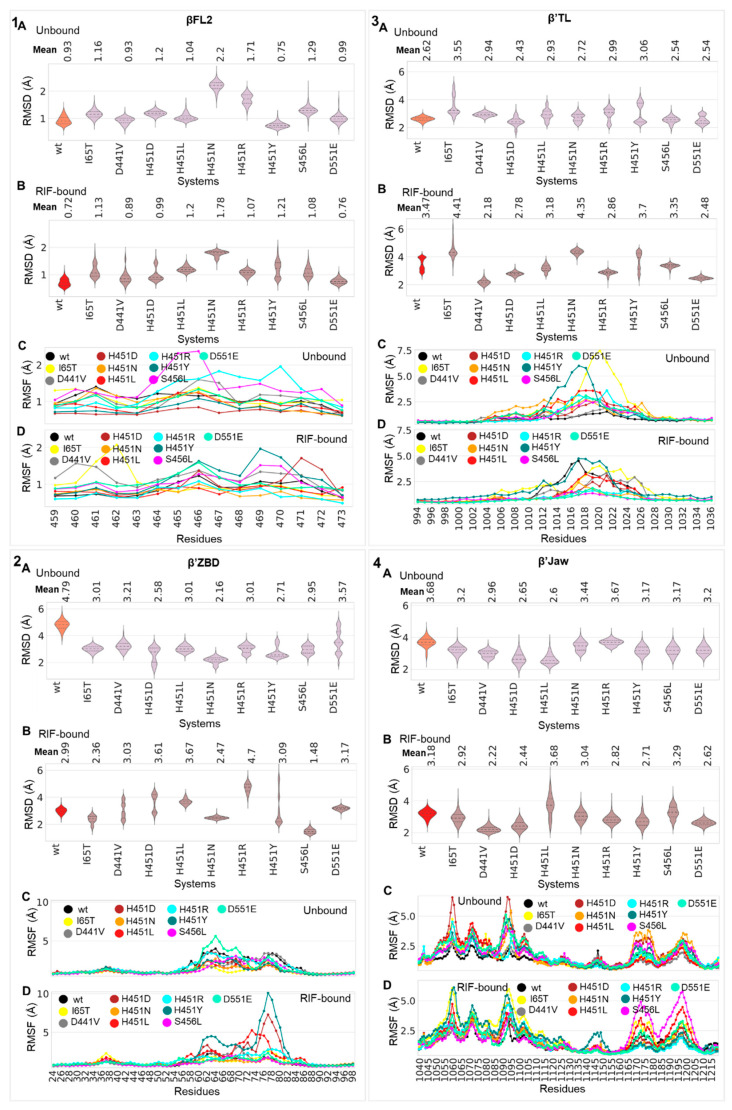
Structural analysis of the *Mtb*-RNAP functional domains; (**1**) βfork loop2 (βFL2), (**2**) β’zinc-binding domain (β’ZBD), (**3**) β’trigger loop (β’TL) and (**4**) β’jaw in *wt* and *mt* systems. Cα-RMSD (with reference to their initial structures) distribution violin plots are shown for the (**A**) unbound and (**B**) RIF-bound *wt*s (unbound, orange; RIF-bound, red) and *mt*s (unbound, pink; RIF-bound, brown). The mean Cα-RMSD values are indicated above the plots while the dotted lines on the violins represent the 25th, 50th and 75th quartiles. The line plots represent the captured Cα-RMSF for (**C**) unbound and (**D**) bound *wt* and *mt Mtb*-RNAP domains.

As seen from Figure 3, the βFL2 domain of the *wt* system exhibited relatively lower Cα-RMSDs for both bound and unbound states while indicating relatively higher Cα-RMSDs in the β’ZBD, β’TL and β’jaw domains. This deduction was further complemented through domain Cα-RMSF, which in some cases indicated correlations between the extent of residue fluctuations and the changes in Cα-RMSD. However, in comparison to the *wt*, the mutant proteins showed varying changes in their respective domains as reflected on the Cα-RMSD and Cα-RMSF data plots. This could, therefore, explain the degree of variations in the stability and flexibility of these regions with respect to RIF binding (Figure 3). Specifically, in the RIF-unbound state we observed that the majority of *mt* βFL2 domains exhibited higher Cα-RMSDs compared to the *wt* with the most notable deviations seen in H451D, S456L, H451R (bimodal) and H451N systems (Figure 3(1A)). In the RIF-bound forms, the *mt*s further displayed higher Cα-RMSDs (especially H451N) with D441V, H451D, S456L and H451Y showing increased conformational sampling (Figure 3(1B)). Cα-RMSD deviations in unbound S456L and H451R and bound I65T and H451Y for instance, may in part be informed by the detected increase in residue fluctuations depicted in Figure 3(1C,D) with particular interest in absolutely conserved βR465 [17] in the unbound systems. As illustrated in Figure 3(2A), all of the unbound *mt*s exhibited a decrease in β’ZBD Cα-RMSD while D551E, H451R, S456L, H451Y, H451D and H451N additionally displayed multiple equilibria. Moreover, large discrepancies in β’ZBD Cα-RMSD for the bound systems were especially observed in H451Y, H451R, H451L, H451D, H451N, I65T and S456L, as shown in Figure 3(2B). Large residue fluctuations between residues 54–84, encompassing the zinc-coordinating β’C60, β’C62, β’C75 and β’C78, which contribute to β’ZBD folding [40], were also especially dominant for H451Y, H451D and H451L and may have largely contributed to the extent of conformational sampling (Figure 3(2D)). Interestingly, all the unbound *mt*s (except S456L and D441V) presented multimodal Cα-RMSD distributions (Figure 3(3A)) for the catalytic β’TL while the I65T, H451R, H451Y, D441V and H451L systems distinctly showed the highest differences in Cα-RMSDs relative to the *wt*. As deduced from Figure 3(3B), a majority of the bound *mt*s exhibited large changes in β’TL Cα-RMSD compared to the *wt* and displayed unimodal distributions. I65T, H451N, H451Y, H451R, H451D, D551E and D441V in particular showed distinct differences in β’TL Cα-RMSD, while large residue fluctuations were prominent between residues 1006–1028 (with β’R1012 and β’H1016 reportedly associated with orienting NTP phosphates [41]) for I65T and H451Y, as similarly observed in the unbound forms (Figure 3(3B–D)). Also indicating high Cα-RMSD differences in the *mt*s relative to the *wt* was the DNA-contacting β’jaw wherein I65T, D551E, S456L, H451Y, D441V, H451D and H451L were especially notable (Figure 3(4A)). As shown in Figure 3(4B), pronounced increases in β’jaw Cα-RMSDs were markedly observed for the bound H451L, H451Y, D551E, H451D and D441V relative to the *wt* system. The observed changes to flexibility in the unbound and bound *mt*s, as depicted in Figure 3(4C,D), especially for H451L and S456L (bound), may have, in part, played a role in the quantified structural deviations, which might suggest an impact on interactions with downstream DNA [42]. Overall, the analysis collectively indicated extensive mutation and RIF-induced perturbation effects on *Mtb*-RNAP’s structural domains, which may potentially affect some enzymatic functions. The β’TL and β’ZBD in particular play important roles in catalysis of transcription [43] and termination and antitermination of transcription [44], respectively. Earlier reports on the diminished capacity of missense RNAP mutants to conduct these catalytic functions [27] make our findings especially interesting and could suggest a link between the observed instabilities and the documented fitness cost [17,45].

### 2.3. Mutational Disruption of the RIF-BP, RIF ‘Active Orientation’ and Binding Affinity

**Figure 4:**
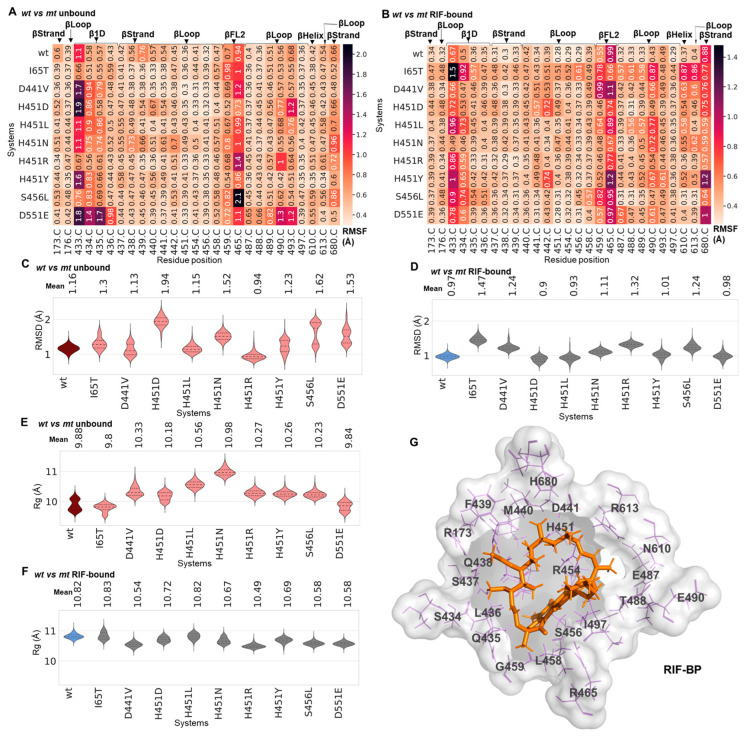
Structural investigation of *Mtb*-RNAP’s RIF-BP dynamics. Cα-RMSF heatmaps of the RIF-BP are illustrated for the (**A**) unbound and (**B**) RIF-bound *wt*s and *mt*s. The heatmaps are accordingly annotated with RMSF estimations wherein low-to-highly fluctuating regions are colored orange to dark purple. Cα-RMSD violin plot distributions are shown for the (**C**) unbound and (**D**) RIF-bound *wt*s (unbound, maroon; RIF-bound, blue) and *mt*s (unbound, orange; RIF-bound, pink), while mean Cα-RMSD values are indicated above the plots. Rg violins are also illustrated for the **(E)** unbound and (**F**) bound systems. The same color scheme was used as in the Cα-RMSD violins. The dotted lines on the violins represent the 25th, 50th and 75th quartiles. (**G**) 3D structure of the annotated RIF-BP (surface representation, grey) with bound RIF (stick representation, orange).

To understand the mechanisms by which these clinically relevant mutations impart on the efficacy of RIF, we elucidated, relative to the *wt*, their structural effects on the dynamics of the RIF-BP correlatively to the ligand dynamics and affinity. Firstly, the dynamics of the RIF-BP was enumerated with the Cα-RMSD and Rg calculations, which helped determine the relative degree of pocket stability or disruptions in the *wt* and *mt* proteins. Center of mass (CoM) calculations were also carried out to monitor changes between the ligand and the RIF-BP. From Supplementary Figure S7A, we observed that the RIF-BP in unbound *wt Mtb*-RNAP exhibited a stable RMSD, while in the *mt* proteins the pocket showed varying magnitudes of perturbations, which were particularly notable in H451D, H451N, H451Y, S456L and D551E. The modes of conformational distribution were further shown in the corresponding violin plots, and accordingly while the *wt* RIF-BP (unbound) was unimodal, the *mt* RIF-BPs majorly exhibited multiple equilibria more apparently in S456L, D551E, H451Y, D441V and I65T (Figure 4C). Taken together, we could deduce a possible correlation between the effects of the mutations and perturbations of the RIF-BP, which entailed key residues such as R173, T433, Q438, F439, D441, P489, N493 and H680 required for mediating active interactions essential for RIF’s stable binding [11,29,31] (Figure 4G). 

The binding of RIF seemed to further stabilize the RIF-BP across the *wt* and *mt* systems (RIF-bound) as evidenced by the relatively lower RMSDs. Pocket perturbations were, however, consistent in some *mt* systems, such as I65T, D441V, H451R and S456L, despite their increased tendencies towards unimodal distributions (Figure 4C). Rg violin distributions revealed that the mutations had considerable effects on the compaction of the RIF-BP more apparently in the unbound state with unimodal distributions and relatively higher Rg values in S456L, H451Y, H451R, H451L and H451N (Figure 4D). I65T, D551E and H451D were, however, distributed similarly to the *wt* protein, which could imply minimal to no effect on the compaction of the pocket. The flexibility of the pocket, however, seemed to increase to varying degrees in the presence of RIF across all systems (except H451N) with effects more apparent in the *wt* and similarly I65T and D551E, which interestingly are distal mutations (Figure 4E). These altogether could indicate changes at the respective RIF-BPs to accommodate RIF.

These RIF-BP discrepancies in the *mt* proteins can be largely attributed to changes in the fluctuations of pocket residues β1D R173, T433 and S434, β loop F439 and βFL2 R465 as seen in the corresponding RMSF data (Figure 4A). Also, β residues T433, S434, R465, E490 and H680 showed notable differences in fluctuation and were the most common across the *mt*s in the presence of RIF (Figure 4B). Changes in side-chain ionic charges in D441V (negative→neutral) and H451Y (positive→neutral) and size in S456L could as well impact on the stable interaction network of the RIF-BP, which, as seen in Figure 4C, was notably perturbed for both mutants. It is also worth noting that *Mtb*-RNAP^I65T^, which presented one of the most notable RIF instabilities, displayed some of the largest fluctuations at β1D T433 and S434, while *Mtb*-RNAP^D551E^ only indicated minimal changes at the same regions, resulting in a more stable yet altered RIF binding. In support, ED calculations revealed increases in RIF-BP sampling in different phase spaces for the *mt*s, for a total captured variance of 80.18% and 5.42% along PC1 and PC2, respectively, (Supplementary Figure S6A) relative to the *wt*. Similarly, for the RIF-bound systems, the *mt*s exhibited higher conformational sampling than the *wt* while highlighting differences in phase space occupancy, as seen both along PC1 (78.56%) and PC2 (5.47%) in Supplementary Figure S6B. Additionally, although the RIF-bound and unbound *mt*s mostly displayed similarities along PC1, they also generally indicated large differences along PC2. 

Our findings further revealed that these characteristic RIF-BP alterations in the *mt*s consequentially affected the binding stability of RIF (Supplementary Figure S7B,C). As seen in the corresponding ligand RMSD plots, RIF displayed highly unstable dynamics in H451N, I65T, H451Y, S456L, H451D, D441V and H451R (Supplementary Figure S7B). Noticeable changes in H451R’s RIF conformational distribution could be due to arginine substituent, which has a longer carbon side chain than histidine, thus making it more difficult for RIF to fit in the pocket and may explain the multimodal equilibria. This effect could also account for the high RIF RMSD in S456L, which acquired a longer leucine substituent. Complementarily, the substitution of histidine in H451R and H451N could affect the formation of ring stabilizing π interactions with RIF hence its unstable conformations in both mutants. On the other hand, RIF exhibited a much more stable RMSD in the *wt* system, which could possibly underlie a stable mode of binding at the RIF-BP. D551E and H451L, however, seemed to be inconsequential on the stability of RIF within the pocket.

**Figure 5:**
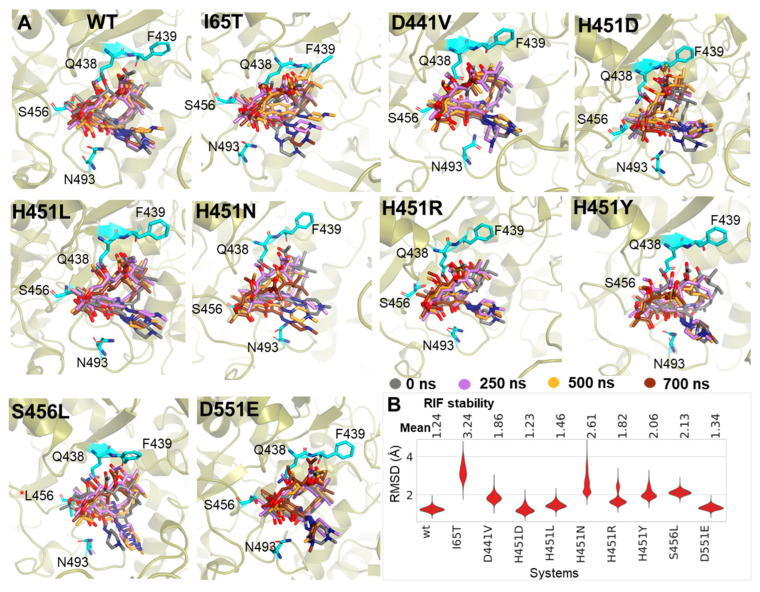
Mutant-induced RIF destabilization. (**A**) 3D illustration of RIF’s shift from the active pose when in the presence of the studied RIF^R^ mutations. RIF snapshots (stick representation) were taken at 0 ns (grey), 250 ns (purple), 500 ns (orange) and 700 ns (brown), respectively. (**B**) The violin plots convey RIF’s stability (with reference to the RIF-BP) for the *wt* and *mt*s over the equilibrated timeframes. The dotted lines on the violins represent the 25th, 50th and 75th quartiles, while mean RMSD values are indicated above the plots.

The impact of the mutations on the binding and stability of RIF can be further explained by the drastic changes to RIF’s hydrogen bond (H-bond) occupancies, which in turn, translated to variations in the active orientation of RIF (Figure 5 and Figure S8A). As previously established, the efficacy of RIF hinges on its native pose within the target pocket to exert the essential steric effects to prevent nascent RNA extension [11]. 

Therefore, the possibility of these mutations to alter this active pose could, in part, contribute to their RIF^R^ mechanisms. While RIF exhibited a stable RMSD of 1.24 Å (with reference to the RIF-BP), its motions increased in the mutants with mean RMSD differences ranging from 0.1 Å to 2.0 Å, which ultimately measures the degree to which RIF moved away from the starting (native) pose (Figure 5B). These changes were also well-informed by the CoM calculations which captured the perturbed motions of RIF away from the RIF-BP (Supplementary Figure S8B). Three-dimensional structural visualization of RIF across the MD simulation (using time-based 3D snapshots) further corroborated the induced destabilization of the active orientation (Figure 5A). As shown, RIF’s orientation at the final timeframe (700 ns) was highly similar to its starting pose pre- and post-equilibrated (0 ns and 250 ns) even though it appeared to move slightly at 500 ns. However, in the mutant systems, varying degrees of orientations were observed at the different timeframes. In other words, while the active pose of RIF appeared to be more conserved in the *wt*, notable alterations were observed in most of the *mt* systems. Consequentially, important interactions [11,29,31], such as H-bonds of O1 and O2 with Q438, O8 with F439, O2 with S456 and O4 with N493, were altered in several cases with the most pronounced changes occurring for I65T, D441V, H451D, H451N, H451Y and S456L where in several instances we observed an increase in lower-frequency H-bond occupancies (Supplementary Figure S8A). Low energy minima structures were used to analyze RIF’s molecular interaction profiles in the *wt* and respective *mt*s. As observed in the *wt*, six H-bonds occurred between β Q438, F439, S456 and N493, which may be important for maintaining RIF’s active pose and stability within the RIF-BP (Supplementary Figure S8A). However, most of the *mt*s displayed different RIF bonding patterns with either the complete loss of any of these important interactions, such as D429 (on σ3.1-3.2 loop) in all the *mt*s, or a change in interaction type as seen in H451R^Q438^ (Supplementary Figure S9).

**Figure 6:**
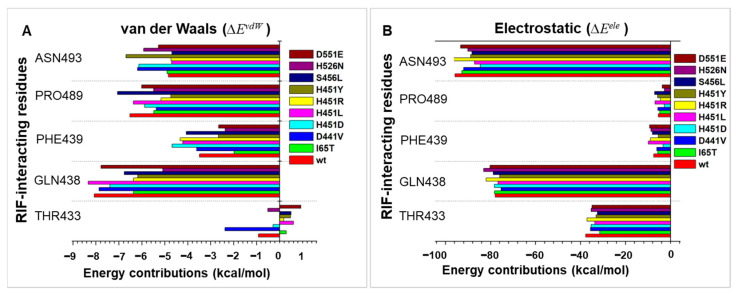
Per-residue decomposition plots showing energy contributions of RIF interacting residues. (**A**) Van der Waals energy decomposition. (**B**) Electrostatic energy decomposition.

Individual energy decomposition of important residues further revealed the impact of the mutations on their contributions to the binding affinity and stability of RIF. Estimations of the relative energetic contributions of these residues further explained the impact of the mutations on the binding of RIF (Figure 6). As shown, electrostatic contributions (Δ*E*^ele^) for F439 reduced from −7.48 kcal/mol in the *wt* to −5.66 kcal/mol in I65T, −6.19 kcal/mol in D441V, −3.34 kcal/mol in H451D and −5.48 kcal/mol in H451Y. Likewise, _wt_F439′s van der Waals contribution (Δ*E*^vdw^ = −3.49 kcal/mol) was reduced in I65T (−1.98 kcal/mol), H451N (−2.36 kcal/mol), H451Y (−2.66 kcal/mol) and D551E (−2.65 kcal/mol). 

Notable reductions in Δ*E*^vdw^ were observed for Q438 with energy differences of −1.71 kcal/mol (I65T), −0.68 kcal/mol (H451D), −2.98 kcal/mol (H451N), −1.70 kcal/mol (H451R), −1.89 kcal/mol (H451Y) and −1.30 kcal/mol (S456L). _S456_Δ*E*^vdw^ reduction was most notable in I65T, which reduced from −5.33 kcal/mol (*wt*) to −4.4 kcal/mol while lowered _S456_ΔE^ele^ differences of −1.53 kcal/mol, −1.05 kcal/mol, −1.43 kcal/mol, −0.82 kcal/mol, −0.37 kcal/mol, −0.65 kcal/mol, −1.5 kcal/mol and −1.26 kcal/mol were observed for I65T, D441V, H451D, H451L, H451N, H451R, H451Y and D551E, respectively. Similarly, for _N493_Δ*E*^ele^ (−95.76 kcal/mol) where the most notable reduction was observed for H451D (−84.67 kcal/mol) and further differences were noted for I65T (−3.04 kcal/mol), D441V (−3.77 kcal/mol), H451L (−8.62 kcal/mol), H451N (−5.66 kcal/mol), H451Y (−6.87 kcal/mol), S456L (−7.49 kcal/mol) and D551E (−2.53 kcal/mol). Complementary decreases in Δ*E*^ele^ and Δ*E*^vdw^ were also observed for T433 and P489, which are also located within the RIF-BP. Taken together, mutation-induced reductions in energy contributions for these essential RIF-binding residues may further impact on the RIF’s affinity and consequentially its inhibitory activity as experimentally reported. This deduction was further supported by the binding energy estimations, which revealed a Δ*G*_bind_ value of −43.58 kcal/mol for RIF in _wt_*Mtb*-RNAP, while binding affinities were reduced in majority of the mutants except H451R, S456L and D551E (Table 1). These relatively higher Δ*G*_binds_ were mainly as a result of the mechanistic re-orientation of RIF’s (away from its active pose) towards more reactive residues, such as R465, where a strong H-bond occurred complementary to the placement of the biphenyl ring (Supplementary Figure S9G,I,J). As earlier detailed, arginine substitution in H451R could have also contributed to its notably high Δ*G*_binds_ in relation to the *wt*. However, we could presume that the change in the active pose of RIF across these three *mt* proteins (as well as others, see Figure 5) is sufficient to limit RIF’s capacity to obstruct RNA extension and enact resistance despite the increased affinity.

### 2.4. Mutation-Associated Destabilization of Catalytic Nucleic Acids Cross-Link to Active RIF’s Disorientation

**Figure 7:**
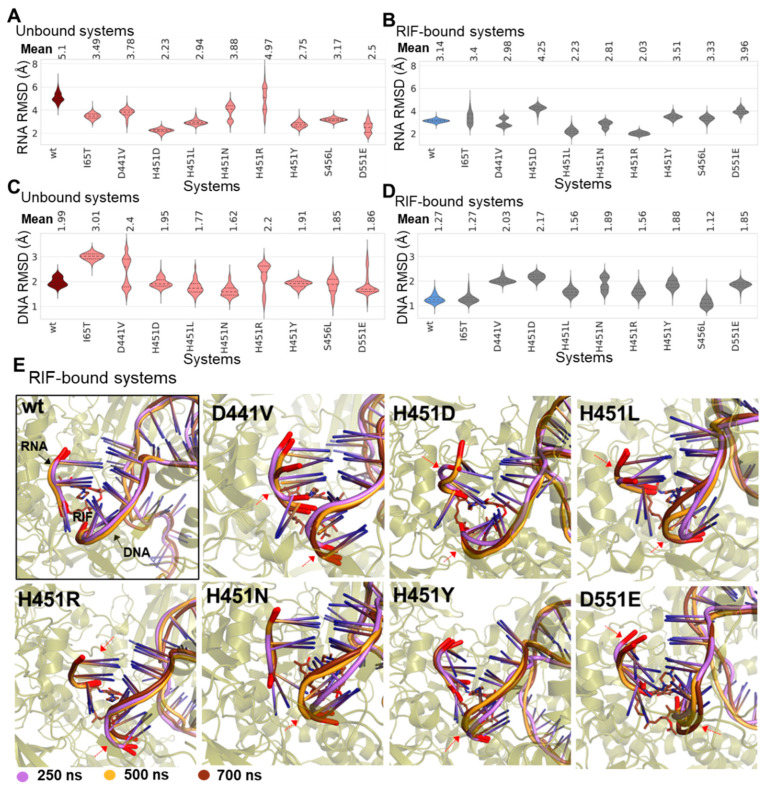
Structure-dynamic analyses of mutational and RIF-binding effects on catalytic nucleic acids. (**A**) Comparative RMSD violin plots for catalytic RNAs in unbound *wt* (maroon) and *mt* (pink) systems and (**B**) catalytic RNAs in RIF-bound protein systems. The mean RMSD values are indicated above the plots, while the dotted lines on the violins represent the 25th, 50th and 75th quartiles. RMSD violin plots for corresponding 4nt DNA moieties are also shown for (**C**) unbound *wt* and *mt* systems and (**D**) RIF-bound proteins. (**E**) Three-dimensional superimposition of the RNA and DNA moieties for the studied systems. Snapshots used were retrieved from 250 ns (colored purple), 500 ns (orange) and 750 ns (brown). Regions with distinctive alterations are indicated with red arrows.

Changes in the dynamics of DNA and RNA may adversely affect the binding efficacy of RIF by altering how effectively the drug interacts with the 5′ end of the RNA transcript to disrupt the active transcription process [11]. This possibility was thus investigated to further provide mechanistic insights on additional mutational effects. Complementary, to the binding dynamics of RIF across the *wt* and *mt* systems, corresponding perturbations at the RIF-interacting RNA and DNA moiety (4nt long 3′ terminal segment of template DNA) were primarily captured using the RMSD metrics. RMSD estimation of RNA deviations in the unbound *wt* revealed a high average RMSD (5.1 Å) relative to its initial position, which was evidently decreased in the presence of RIF (3.14 Å) (Figure 7A,B).

The same trend was also observed for the DNA moiety suggesting a motional restriction induced by the presence of RIF. This was further corroborated by the consistency observed in superimposed snapshots (from specific timeframes) of RNAs and the DNA moieties in the RIF-bound *wt* as illustrated in Figure 7E. The unbound *mt*s (except H451R), however, showed lowered RNA RMSDs when compared to the unbound *wt*. Furthermore, a majority of the unbound *mt*s indicated large RMSD discrepancies for the DNA moiety, particularly in H451N, H451L, S456L D551E, H451R and D441V, which together with the RNA observations may contribute to mutation-induced inefficiencies in *Mtb*-RNAP’s processivity. Interestingly, most of the *mt*s displayed more unstable RNA molecules as informed by the average RMSDs (Figure 7A,B). Also, although most of the RIF-unbound *mt*s showed lowered RMSDs for the DNA moiety (Figure 7C), the RIF-bound *mt*s generally indicated greater instabilities as captured in Figure 7D.

## 3. Materials and Methods

### 3.1. Data Retrieval and Protein Preparation

The 3D crystal structure of the *Mtb*-RNAP was retrieved from the Protein Data Bank (PDB:5UHC) [11]. Also obtained was the 3D structure of *Thermus thermophilus* (*Tth*) RNAP (PDB:1ZYR) [46], which was used as a partial template to model the missing residues in the target protein. Redundant crystal molecules not of interest to this study were removed to obtain a multi-subunit *Mtb*-RNAP structure with the αI-NTD and αII-NTD, β and β’ subunits, ω and σ subunits; DNA, RNA and RIF molecules; and Mg^2+^ and Zn^2+^ ions. More so, high-confidence mutations investigated in this study were selected based on their recurrence in clinical studies conducted globally as reported in literature [47] as well as data deposited in MUBII-TB-DB [33], and they include D441V, H451D, H451L, H451N, H451R, H451Y, S456L [33] and D551E [34]. Also considered was the novel *Mtb*-RNAP I65T mutation recently implicated in RIF-resistance.

### 3.2. Structural Modelling of Wildtype (wt) and Mutant (mt) Mtb-RNAPs

The MODELLER software (v9.15) (University of California, San Francisco, CA, USA) [48] was used to remodel the missing regions in the crystallized *Mtb*-RNAP, which are the αII-NTD (residues 156–157) and the β’TL (residues 1012–1025), using homologous *Tth*-RNAP as the structural template. This approach was employed to model the 3D structures of the *wt* and *mt* RNAPs via a very slow refinement option to give a total of 100 models for each, which were ranked based on the normalized DOPE score (z-DOPE score). Prior to structural modelling, point substitutions were carried out in the target sequence to obtain each of the *Mtb*-RNAP *mt*s of interest in this study. The best structural models for the *wt* and *mt*s were ultimately selected based on the lowest z-DOPE scores, while PROCHECK (University of California, Los Angeles, CA, USA) [49] and QMEAN (University of Basel, Basel, Switzerland) [50] quality assessment servers were used to further validate model quality (Supplementary Table S1). This modelling method is efficient and would not introduce errors in the generated models compared to the crystal structure as previously reported [36]. Furthermore, to obtain the unbound *Mtb*-RNAP multi-subunit systems for each *wt* and *mt*s, RIF was removed from the corresponding model that was generated prior to MD simulations.

### 3.3. Molecular Dynamics (MD) Simulations and RIF Affinity Determination

The GROMACS 2016.1 software (The Pennsylvania State University, State College, PA, USA) [51] was used to conduct 700ns MD simulations on the *Mtb*-RNAP protein multi-subunit studied herein. Specifically, these entail the unbound *wt*s and 9 *mt* (I65T, D441V, H451D, H451L, H451N, H451R, H451Y, S456L and D551E) proteins as well as their respective RIF-bound forms making a total of 22 systems. Simulations were performed on the Center for High Performance Computing (CHPC) clusters in Cape Town. Each *Mtb*-RNAP (both unbound and RIF-bound) system employed for our simulation study uniformly consisted of the αI-NTD and αII-NTD, β and β’ subunits, ω and σ subunits; DNA and RNA molecules; and Mg^2+^ and Zn^2+^ ions. The simulations were carried out using Amber ff99SB-ILDN [52] with a triclinic box and a clearance space of 1.6 nm. The system was solvated using the rigid extended simple point charge (SPC/E) water model, and the protein’s overall charge was neutralized with Na^+^ and Cl^−^ counterions. RIF’s topology parameters were generated using the Antechamber Python Parser Interface (ACPYPE) 0.1.1 tool (European Bioinformatics Institute, Cambridge, UK) [53] following valency corrections using Discovery studio 4.5 [54]. The systems were minimized using up to 50,000 steps of steepest-descent and terminated when the maximum force was <1000 kJ mol^−1^ nm^−1^. The short-range cut-offs for the non-bonded interactions were set to 1.3 nm. The long-range interactions were treated using the Particle Mesh Ewald (PME) algorithm by applying a grid spacing of 0.16 nm and a cut-off distance of 1 nm while using the LINCS method to constrain all the bond lengths. The systems were then temperature and pressure equilibrated in the NVT ensemble with V-rescale coupling set to 300 K, and an NPT ensemble with Parrinello–Rahman barostat set to 1 bar for 100 ps, respectively. Moreover, a duplicate run was performed with the *wt* RNAP multi-subunit as the control sample to ensure a high degree of correctiveness and reproducibility. This was also important to ascertain that the conformational changes observed in the mutants post-equilibration are largely due to mutational effects.

Resulting trajectories were corrected for periodic boundary conditions using *gmx trjconv,* while conformational analyses were performed with the *gmx rms*, *gmx rmsf*, *gmx gyrate* and *gmx hbond* tools which, respectively, were used to calculate RMSD, Cα-RMSF, Rg and quantify H-bond interactions. Additionally, the *readHBmap.py* script (https://github.com/quytruong1808/vilas/blob/master/vilas/analyzer/readHBmap.py (accessed on 20 July 2021)) was used for H-bond analyses. All versus all Cα-RMSD was calculated using a previously applied in-house script [55] that implemented the pytraj package [56] at a step size of 20 frames. The script processed each of the trajectories and then calculated the Cα-RMSD via comparison of each MD frame to all other frames and itself across the simulation time to further corroborate the stability of the control systems. Additionally, to analyze events at the RIF-BP, the gmx select tool was used to map out *wt* β subunit residues within 5Å of RIF, among which include R173, R176, F439, Q435, Q438, D441, H451, R454, S456 and R465, for the initial frame of the simulation. Motional deviations of RIF across the *wt* and *mt* systems relative to the RIF-BP were also determined via CoM analysis using the *gmx distance* tool. The VMD software (v1.9.3) (University of Illinois, Urbana-Champaign, IL, USA) [57] was used for structural visualization of the MDs. Graphical analyses and visualizations were further done using RStudio and a combination of Python packages, namely, pandas [58], seaborn [59], NumPy [60] and Matplotlib [61].

Furthermore, affinity of RIF for the *wt* and *mt* β subunits were evaluated through Molecular Mechanics/Generalized-Born Surface Area (MM/GBSA) calculations using the gmx_MM/PB(GB)SA tool [62]. The MM/GBSA calculations were done with the final 10 ns (5000 timeframes) of the trajectories and making use of the Amber ff99SB-ILDN [52] force field. These equilibrated timeframes were selected for the energy calculations to minimize possible entropical effects.

### 3.4. Comparative Essential Dynamics Analysis

ED calculations with common principal components (PCs) (also referred to as orthogonal eigenvectors) attained through a single decomposition of multiple MD trajectories was conducted using the comparative ED MDM-TASK-web tool (Rhodes University, Makhanda, South Africa) [63,64] (https://github.com/RUBi-ZA/MODE-TASK/tree/mdm-task-web (accessed on 20 September 2022)). The tool was used to process unbound and RIF-bound *wt* and *mt Mtb*-RNAP trajectories to produce decomposed conformational data in scatter plot representation with common PCs. Given that PC1 and PC2 captures the most variance, they were used in the analysis. The following Cα residue selections were made to improve resolution of the *Mtb*-RNAP ED results: ‘(chainid 0 and residue 7 to 221) or (chainid 1 and residue 228 to 444) or (chainid 2 and residue 482 to 1562) or (chainid 3 and residue 1584 to 2850) or (chainid 4 and residue 2873 to 2940) or (chainid 5 and residue 2945 to 3262)’. These selections correspond to chain A residues 9 to 223, chain B residues 9 to 225, chain C residues 58 to 1138, chain D residues 8 to 1278, chain E residues 40 to 107 and chain F residues 210 to 527, respectively. A step size of 5 frames was further used in the *Mtb*-RNAP EDs while additional RIF-BP ED calculations were conducted using step size of 1 frame.

## 4. Conclusions

TB continues to rank as one of the leading causes of mortality in the modern world, and although RIF remains one of the more potent of the first-line drugs against TB, the prevalence of RR-TB and MDR-TB continues to grow. This global threat, therefore, needs to be curbed by designing or discovering drugs that can overcome the challenges posed by anti-tubercular drug resistance. Hence, our study attempted to elucidate the mechanism of resistance of clinically relevant RIF-proximal and distal missense mutations at the molecular level. To do this, the *wt* and *mt Mtb*-RNAPs were first modelled and applied to MD simulations. Following post-MD preparations, traditional trajectory analysis approaches, namely CoM, RMSD, RMSF, Rg, H-bond occupancy and a recently developed comparative ED approach [63] were applied. Additionally, the binding free energies of RIF towards the β subunit were determined through the MM/GBSA and per-residue decomposition approaches [62].

The following are the key observations made: (I) The mutated RNAPs generally resulted in increased conformational sampling with increased flexibility in various loop regions across *Mtb*-RNAP showing that the mutations elicited a global motional effect. These changes were further linked to altered structural domain flexibilities, which may be important in the *wt* for the associated critical functions such as inherent stability, termination capacity and overall processivity, that tend to be diminished in *mt*s as reported in literature [27]. The heightened motions may, therefore, be a compensatory mechanism required for resistance. (II) The active RIF pose shifted by varying degrees in the presence of the *mt*s, including mutations with increased binding affinity, further suggesting that RIF efficacy may rely more on its active pose to elicit its inhibitory effects. (III) We further found that besides RIF-proximal missense mutations such as D441V, which can affect RIF binding affinity or pose by directly affecting RIF-BP stability, distal mutations, such as I65T, can still greatly destabilize RIF’s active pose by introducing instability to structural domains, such as the β1D, which forms part of the RIF-BP. (IV) Finally, it was especially interesting to note that some resistance mechanisms may rely on the destabilization of catalytic DNA and/or RNA to overcome RIF’s inhibitory effects. Overall, our findings gave an informed perspective to the effects of missense mutations on the structure and function of *Mtb*-RNAP and may be informative for future drug design or discovery protocols.

## Figures and Tables

**Table 1:** The relative binding affinities of RIF against the *wt* and *mt Mtb*-RNAPs.

Systems	Δ*E*^vdW^ (kcal/mol)	Δ*E*^ele^ (kcal/mol)	Δ*E*^gb^ (kcal/mol)	Δ*E*^surf^ (kcal/mol)	Δ*G*^gas^ (kcal/mol)	Δ*G*^solv^ (kcal/mol)	Δ*G*^bind^ (kcal/mol)
*wt*	−62.9 ± 3.3	−32.5 ± 3.4	59.0 ± 1.0	−7.2 ± 0.07	−95.4 ± 4.8	51.8 ± 1.1	−43.6 ± 4.9
I65T	−40.6 ± 3.5	−26.9 ± 5.7	52.3 ± 4.0	−5.5 ± 0.04	−67.6 ± 6.7	46.8 ± 4.0	−20.8 ± 7.8
D441V	−58.0 ± 3.3	−15.3 ± 2.6	46.1 ± 1.3	−7.0 ± 0.05	−73.2 ± 4.2	39.1 ± 1.3	−34.1 ± 4.4
H451D	−63.0 ± 3.3	−24.5 ± 4.8	55.6 ± 1.9	−7.6 ± 0.05	−87.6 ± 5.8	48.0 ± 1.9	−39.5 ± 6.1
H451L	−57.2 ± 3.5	−44.3 ± 2.3	66.3 ± 0.8	−6.9 ±0.06	−101.5 ± 4.2	59.4 ± 0.8	−42.1 ± 4.2
H451N	−45.5 ± 3.0	−30.3 ± 4.6	49.2 ± 2.8	−5.2 ± 0.06	−75.8 ± 5.6	44.0 ± 2.8	−31.8 ± 6.2
H451R	−60.0 ± 3.3	−65.3 ± 3.0	82.1 ± 1.4	−7.3 ± 0.04	−125.3 ± 4.5	74.8 ± 1.4	−50.5 ± 4.7
H451Y	−55.4 ± 3.0	−30.5 ± 1.9	56.3 ± 0.9	−6.9 ± 0.05	−85.9 ± 3.6	49.5 ± 0.9	−36.4 ± 3.7
S456L	−64.2 ± 3.5	−43.2 ± 3.7	71.4 ± 1.7	−7.7 ± 0.01	−107.3 ± 5.1	63.7 ± 1.7	−43.6 ± 5.4
D551E	−67.4 ± 3.3	−55.3 ± 3.6	77.3 ± 1.9	−8.8 ± 0.1	−122.7 ± 4.9	68.6 ± 1.9	−54.1 ± 5.2

Δ*E*^vdW^—van der Waals energy, Δ*E*^ele^—electrostatic energy, Δ*E*^gb^—polar contribution to solvation free energy, Δ*E*^surf^—non-polar contribution to solvation free energy, Δ*G*^gas^—gas phase energy, Δ*G*^solv^—solvation free energy and Δ*G*^bind^—total binding energy.

## Data Availability

Data produced in the present study is included in this article and in the Supplementary Materials. Homology models and the molecular dynamics trajectories may be requested from the corresponding author if required.

## Supplementary Materials

The following supporting information can be downloaded at: https://www.mdpi.com/article/10.3390/ijms24043313/s1.

## Abbreviations

CoM	Center of mass
ED	Essential dynamics
MD	Molecular dynamics
*mt*	Mutant
*Mtb*	*Mycobacterium tuberculosis*
Rg	Radius of gyration
RIF	Rifampicin
RNAP	RNA polymerase
RMSD	Root mean square deviation
RMSF	Root mean square fluctuation
RRDR	RIF-resistance-determining region
*wt*	Wild type
