# Supplementary material for: Investigation of Multi-Subunit Mycobacterium tuberculosis DNA-Directed RNA Polymerase and Its Rifampicin Resistant Mutants
Source: Int J Mol Sci. 2023 Feb 7;24(4):3313. doi: 10.3390/ijms24043313 (PMC9965755; doi:10.3390/ijms24043313)
Supplement: Supplementary file 1 [file ijms-24-03313-s001.zip › ijms-2135285-supplementary.pdf]

International Journal of *Molecular Sciences*

Investigation of multi-subunit *Mycobacterium tuberculosis* DNA-directed RNA polymerase and its rifampicin resistant mutants

Supplementary information

Mokgerwa Zacharia Monama [1], Fisayo Olotu [1] and Özlem Tastan Bishop [1,*]

[1] Research Unit in Bioinformatics (RUBi), Department of Biochemistry and Microbiology, Rhodes University, Makhanda 6139, South Africa
* Correspondence: o.tastanbishop@ru.ac.za

Table S1. Summary of homology modelling and validation results using z-DOPE, QMEAN, PROCHECK and RMSD.

Systems	z-DOPE score (MODELLER)	RMSD (Å) (5uhc *vs* model)	QMEAN6 (normalized score)	PROCHECK (Ramachandran plot)
**wt**	-0.52	0.21	0.71	Favored, 94.1%; unfavored, 0.1%
**I65T**	-0.52	0.20	0.72	Favored, 94.1%; unfavored, 0.1%
**D441V**	-0.52	0.20	0.71	Favored, 94.0%; unfavored, 0.1%
**H451D**	-0.52	0.21	0.71	Favored, 93.9%; unfavored, 0.1%
**H451L**	-0.52	0.20	0.71	Favored, 94.0%; unfavored, 0.1%
**H451N**	-0.52	0.21	0.71	Favored, 93.8%; unfavored, 0.1%
**H451R**	-0.52	0.22	0.71	Favored, 93.8%; unfavored, 0.1%
**H451Y**	-0.51	0.21	0.71	Favored, 93.8%; unfavored, 0.1%
**S456L**	-0.51	0.21	0.71	Favored, 93.6%; unfavored, 0.1%
**D551E**	-0.52	0.21	0.71	Favored, 93.9%; unfavored, 0.1%

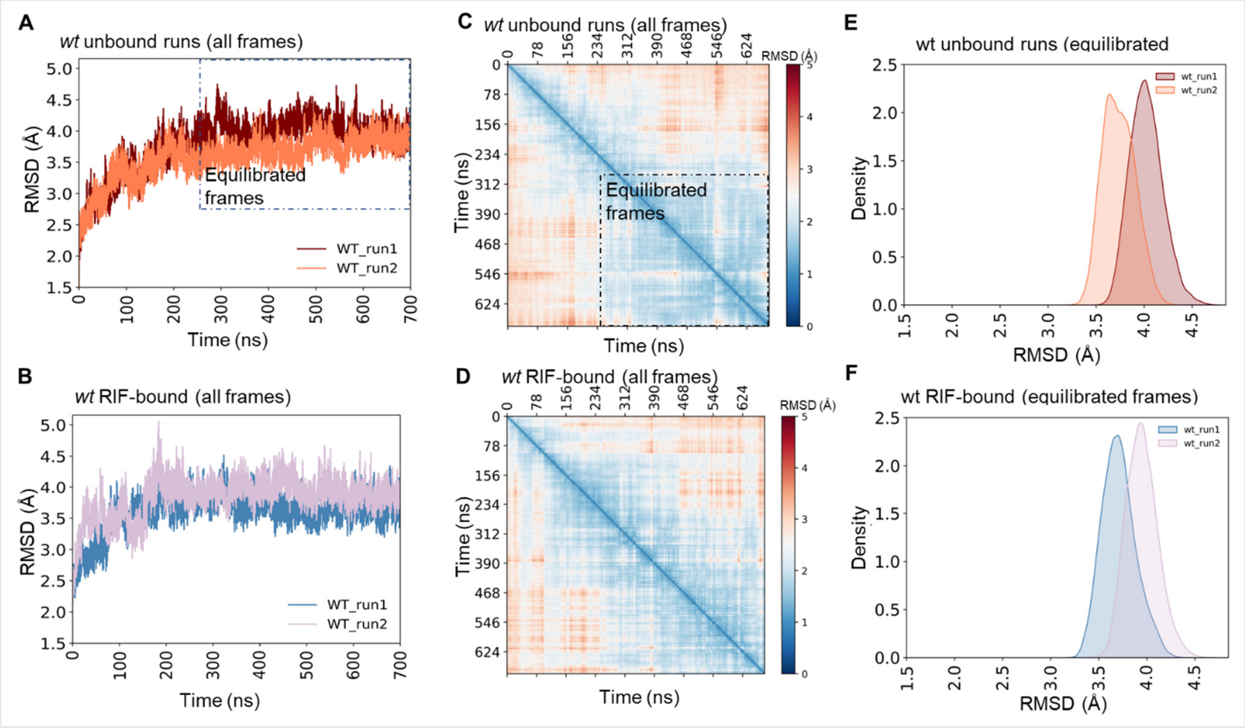

Figure S1. Graphical representation of the structural agreement between the duplicate *wt* runs over the equilibrated timeframes**. (A-B)** The line plots represent the Cα-RMSD of *wt* run1 (unbound, maroon; RIF-bound, blue) and *wt* run2 (unbound, orange; RIF-bound, pink), with respect to their initial structures. All versus all Cα-RMSD heatmaps are also shown for the **(C)** unbound and **(D)** RIF-bound *wt* run1 systems respectively. The heatmaps are colored from red (conformationally dissimilar) to blue (conformationally similar). Kernel density estimation (KDE) plots were are shown for the **(E)** unbound and **(F)** RIF-bound duplicate *wt* runs for the equilibrated timeframes. A similar colour scheme used in the line plots is used for the KDEs.

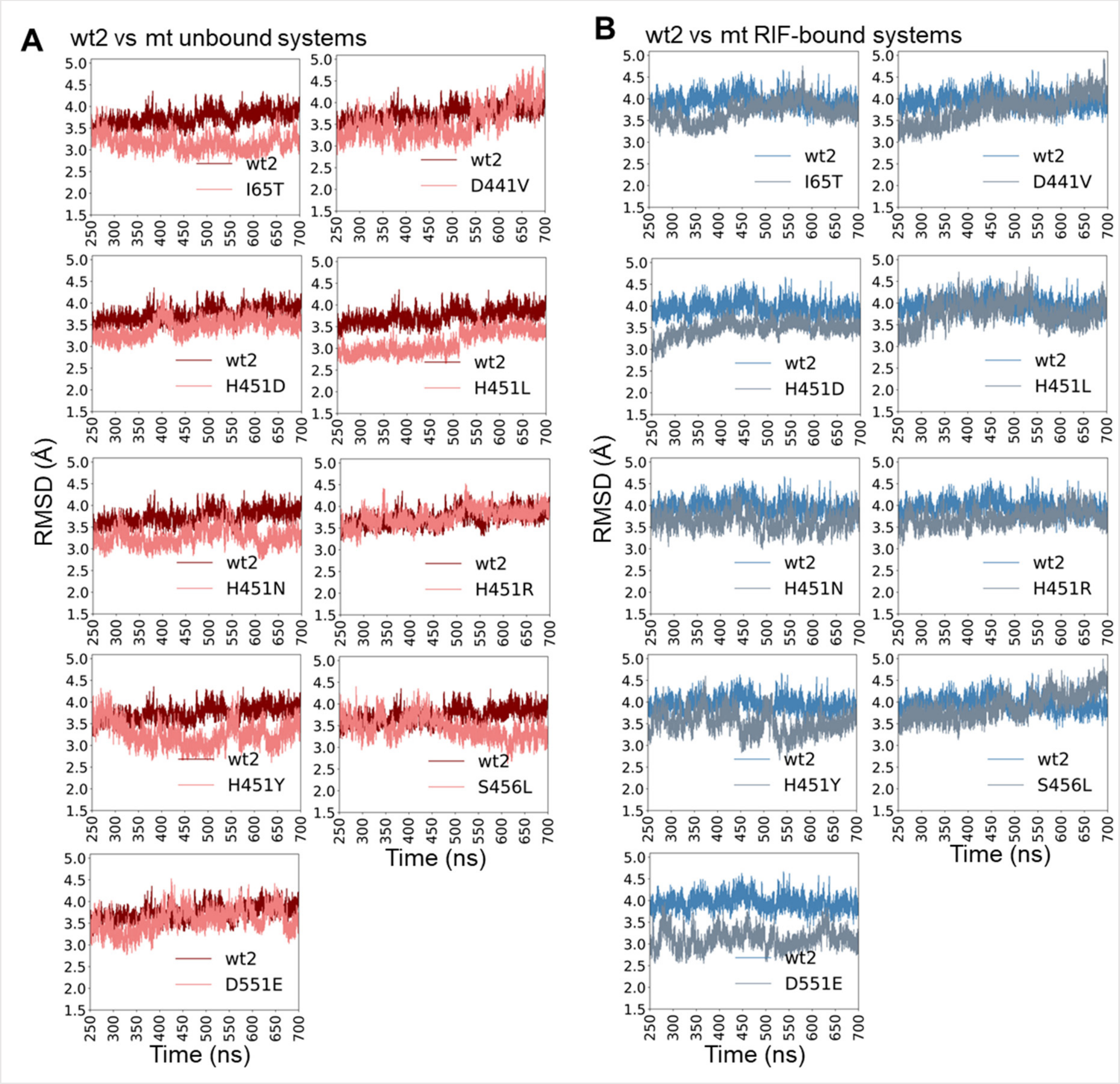

Figure S2. Graphical representation of the mutation- and/or RIF-induced effects on *Mtb*-RNAP conformational stability**.** The line plots represent the estimated Cα-RMSD comparison of the *wt* (unbound, maroon; RIF-bound, blue) and *mt*s (unbound, orange; RIF-bound, pink) for the **(A)** unbound and **(B)** bound proteins over time, with respect to their initial structures.

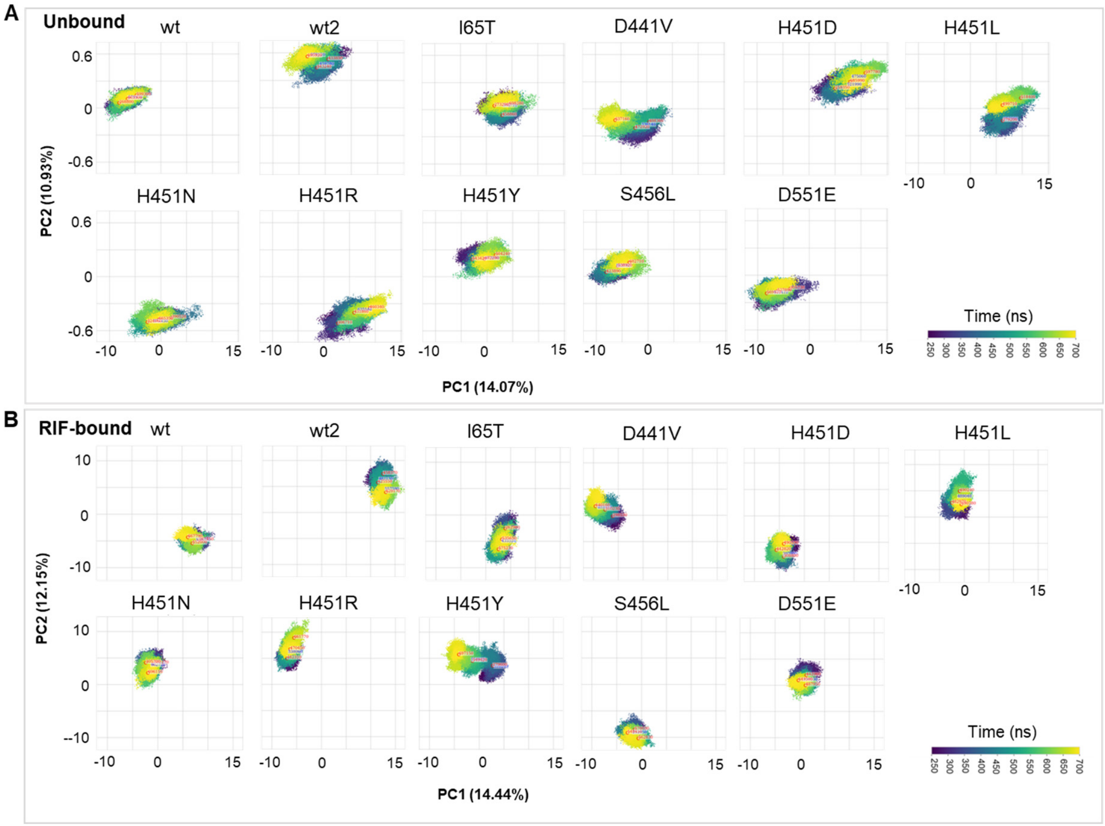

Figure S3. Comparative essential dynamics plots of the (**A**) unbound and (**B**) RIF-bound *wt* and *mt Mtb*-RNAPs. Each dot represents a conformation, and the dots are colored according to the time of sampling (dark to yellow).

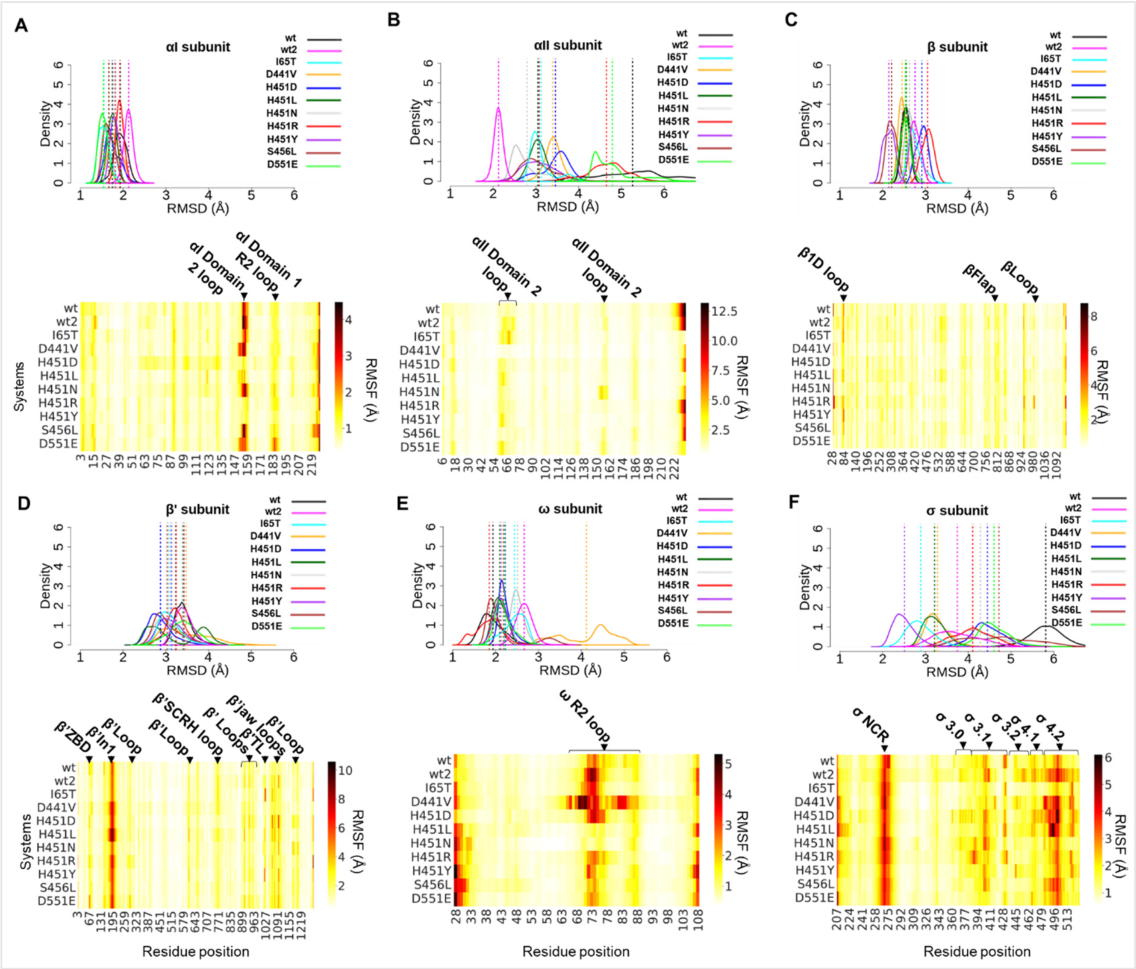

Figure S4. Graphical representation of the *Mtb*-RNAP subunit behavioral comparison between the *wt* and *mt*s for the unbound proteins**. (A-F, Top)** The KDE plots show the extent of conformational sampling as determined by Cα-RMSD (Å) for the *wt* and *mt* RNAP subunits, relative to their initial structures. **(A-F, Bottom)** The heatmaps represent the respective subunit Cα-RMSF (Å) readings for each protein, colored yellow (less residue fluctuations) to black (most residue fluctuations). The annotations indicate regions with the most contribution towards changes in subunit Cα-deviations.

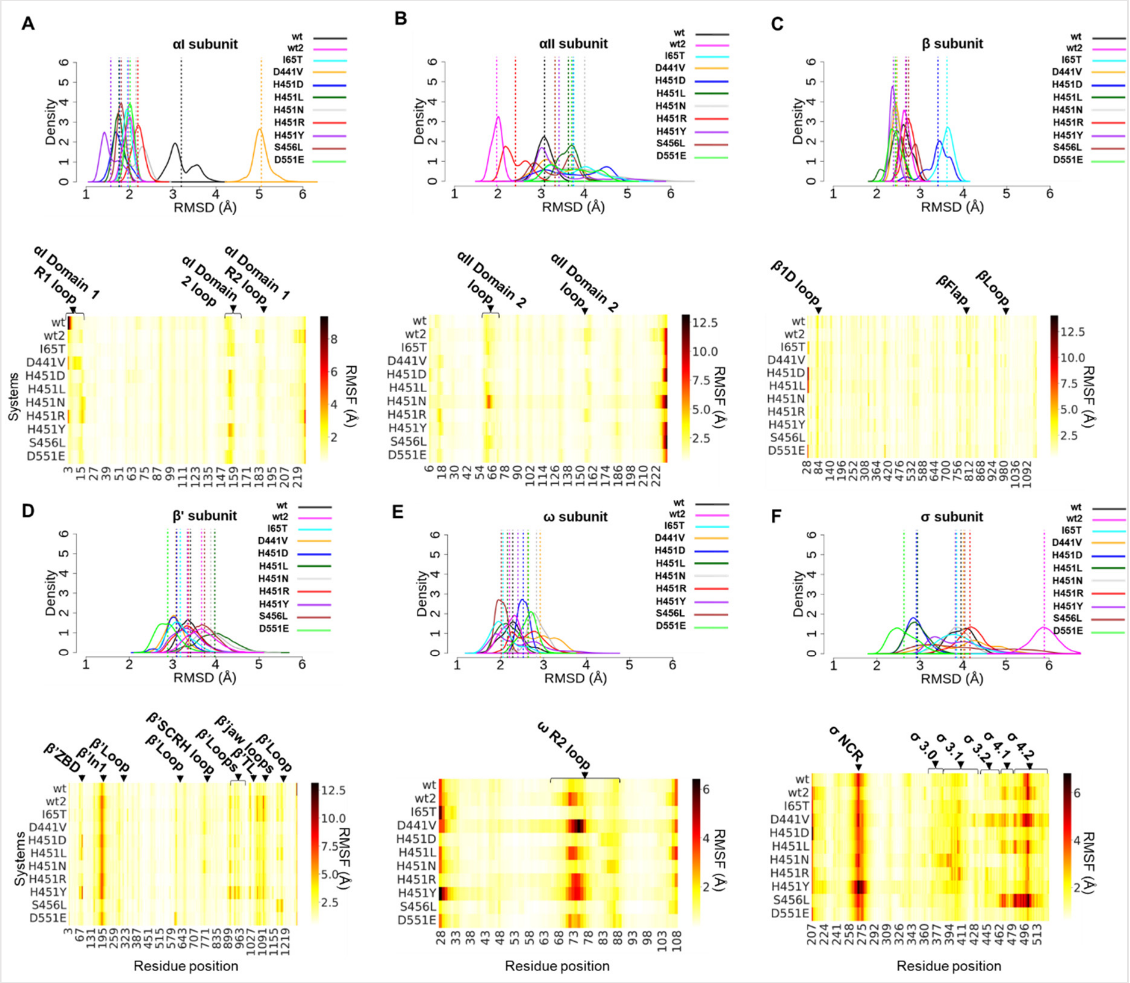

Figure S5. Graphical representation of the *Mtb*-RNAP subunit behavioral comparison between the *wt* and *mt*s for the RIF-bound proteins. **(A-F, Top)** The KDE plots show the extent of conformational sampling as determined by Cα-RMSD (Å) for the *wt* and *mt* RNAP subunits, relative to their initial structures. **(A-F, Bottom)** The heatmaps represent the respective subunit Cα-RMSF (Å) readings for each protein, colored yellow (less residue fluctuations) to black (most residue fluctuations). The annotations indicate regions with the most contribution towards changes in subunit Cα-deviations.

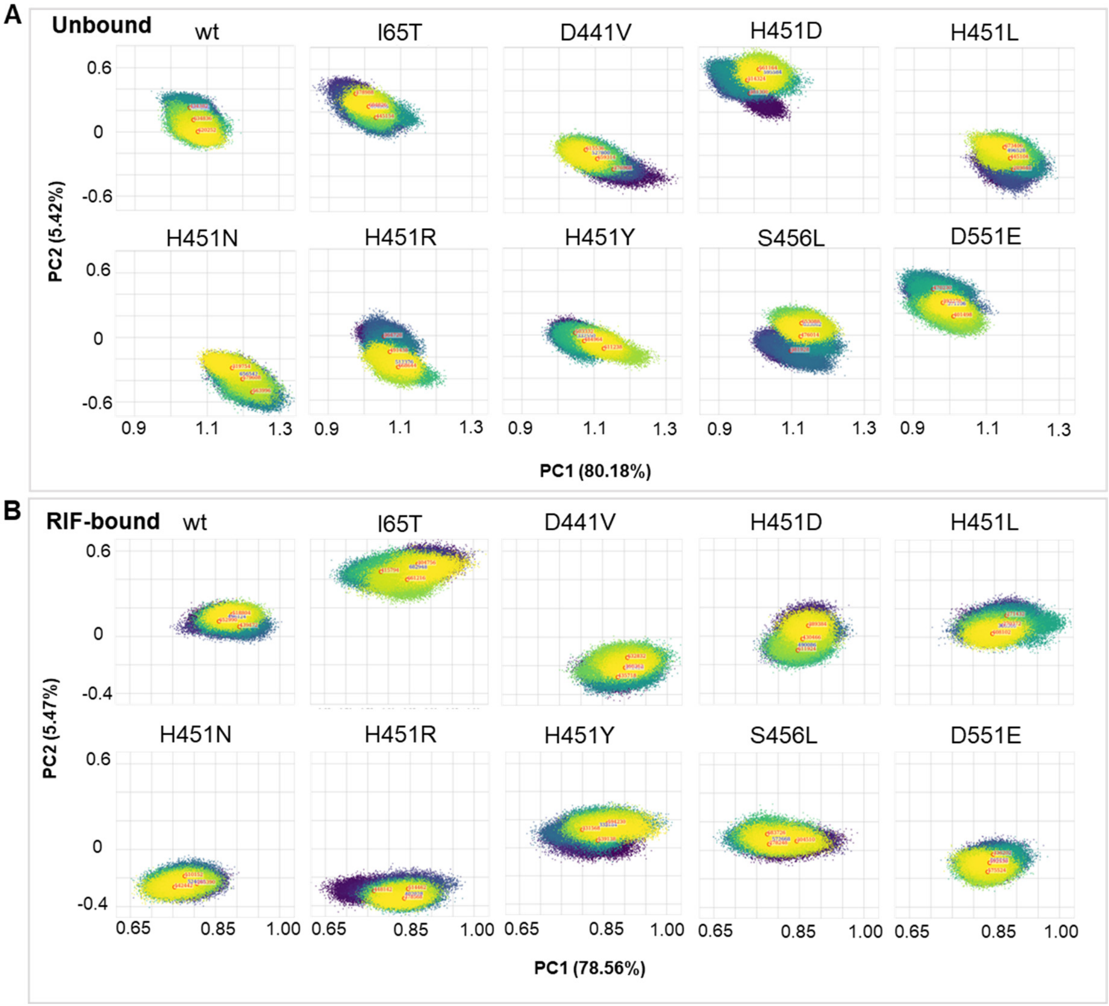

Figure S6. Comparative essential dynamics plots of the (**A**) unbound and (**B**) RIF-bound *wt* and *mt* RIF-BPs**.** Each dot represents a conformation, and the dots are colored according to the time of sampling (dark to yellow).

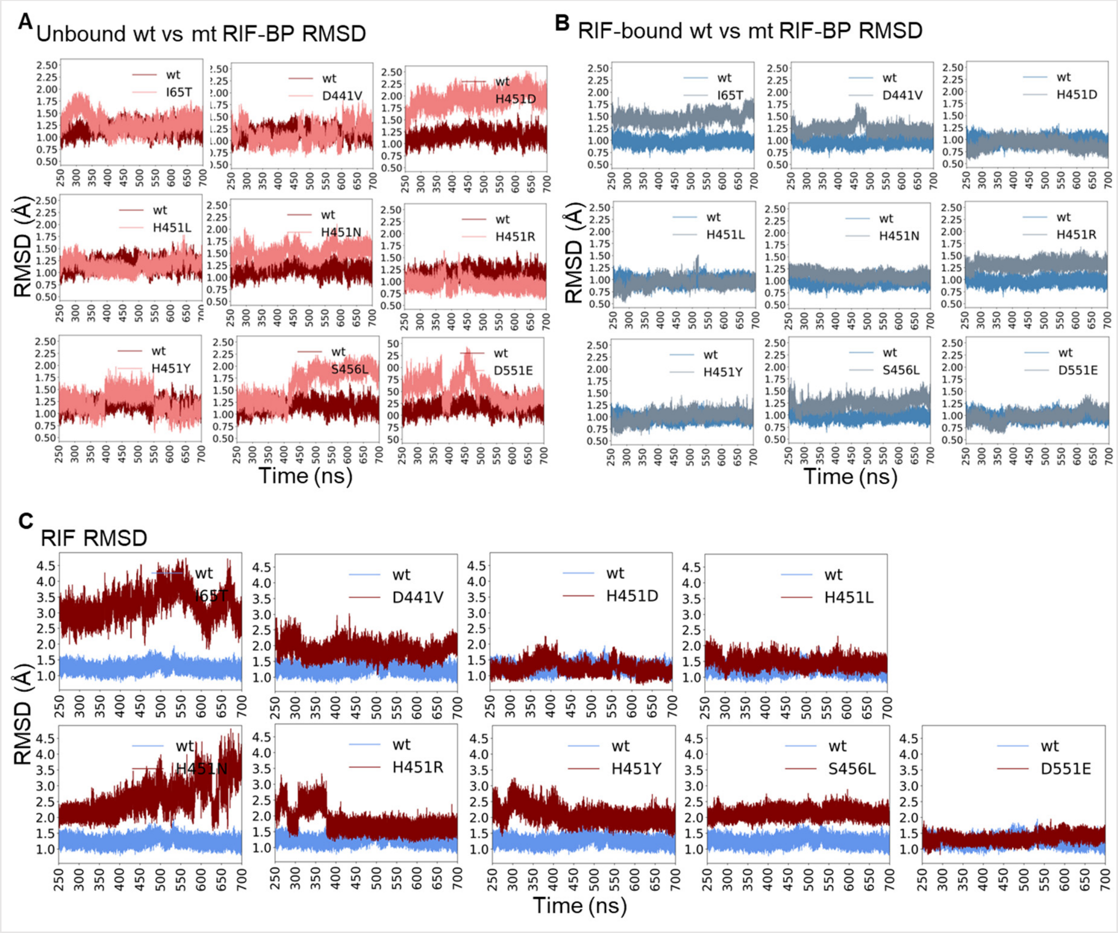

Figure S7. Graphical representation of RIF and RIF-BP stability over the course of the simulation**.** The estimated Cα-RMSD RIF-BP line plots illustrate the differences between of the *wt* (unbound, maroon; RIF-bound, blue) and *mts* (unbound, orange; RIF-bound, pink) in the **(A)** unbound and **(B)** RIF-bound forms over time, with respect to their initial structures. **(C)** The RMSD line plots for RIF detail the stability of the ligand (with respect to the RIF-BP) for the *wt* (blue) and *mt*s (maroon) over the course of the simulations.

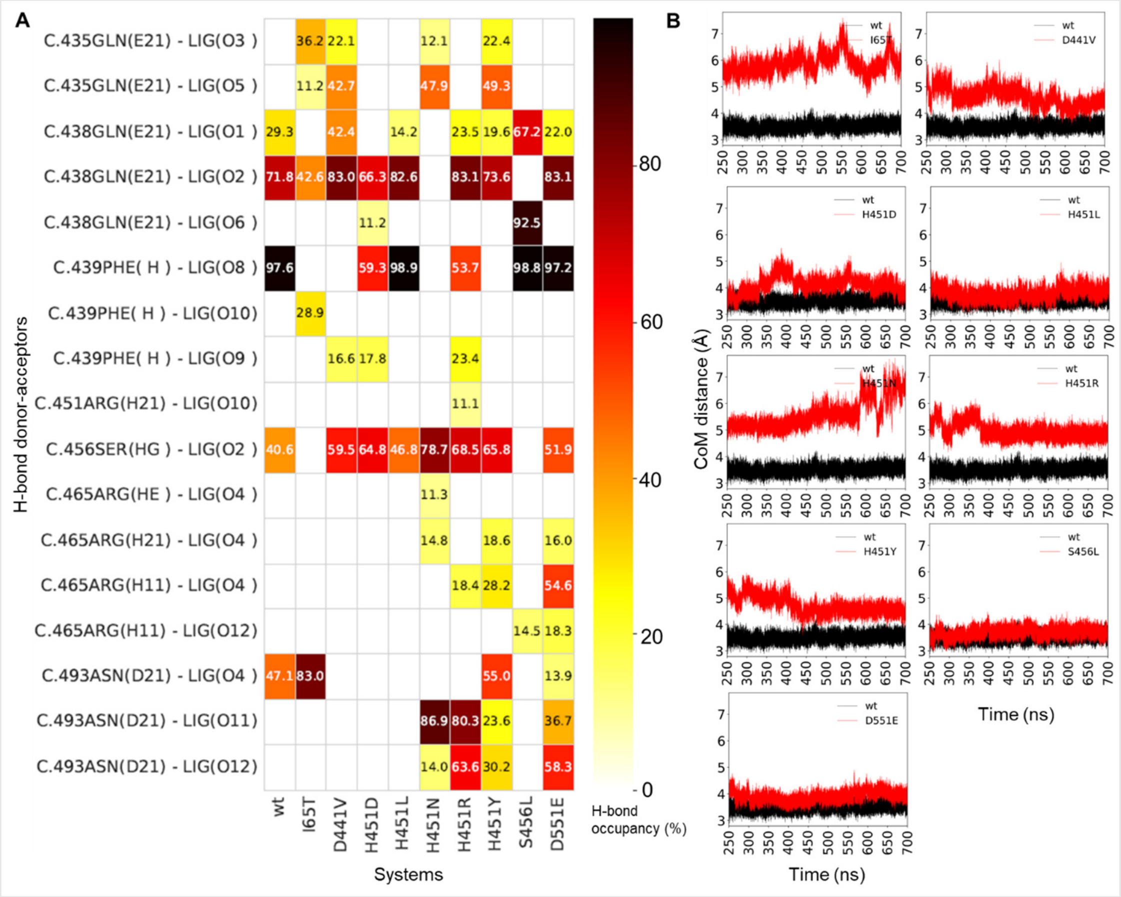

Figure S8. Graphical representation of RIF's H-bond interactions and displacements over time. **(A)** H-bond occupancy profile (frequency of H-bond interactions) for the *wt* and respective *mts*. **(B)** CoM plots represent the change in RIF's distance (Å) from the RIF-BP over the captured timeframes.

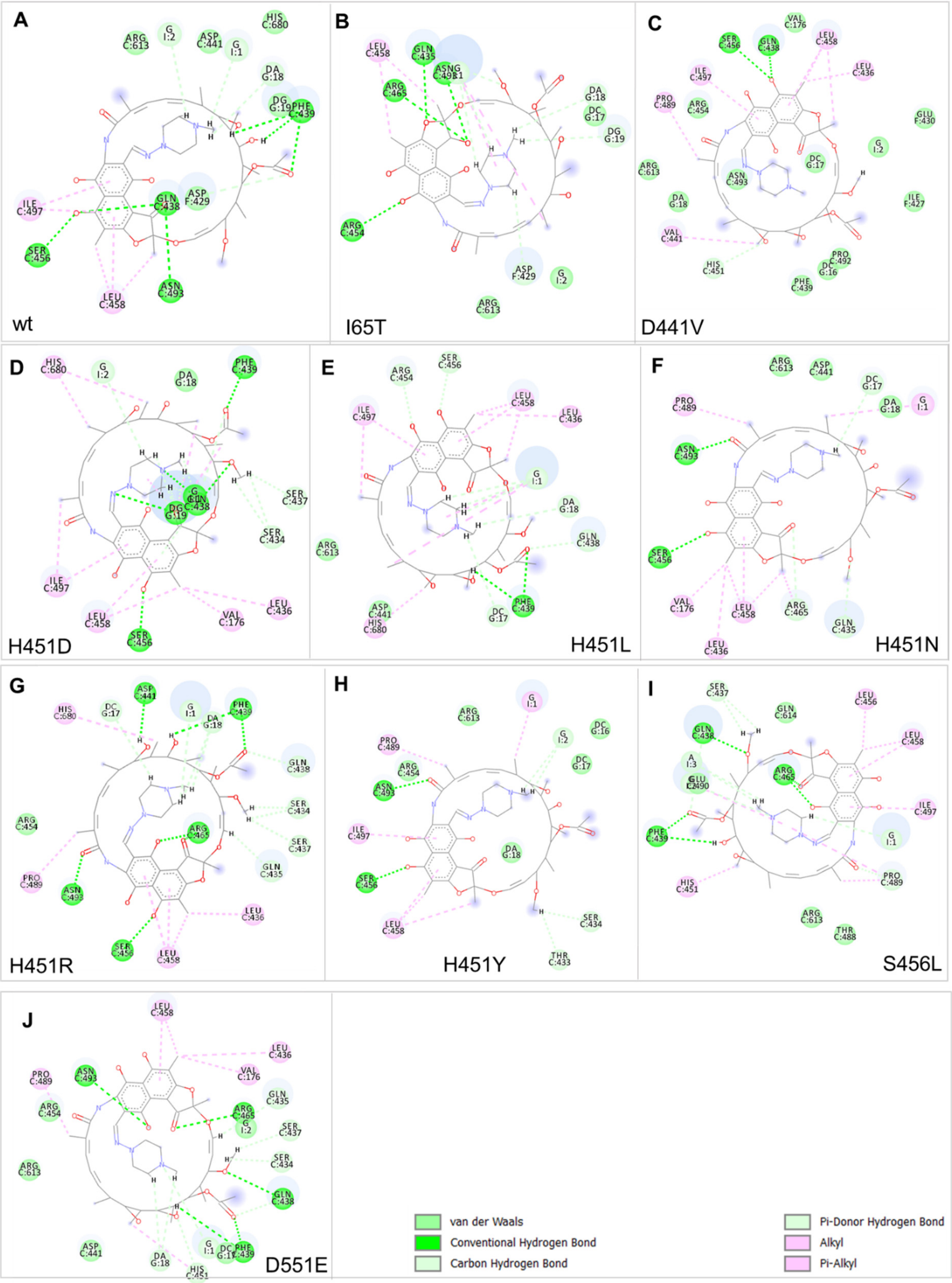

Figure S9. (A-J) RIF interaction profiles of the low energy minima RIF-BP structures for the respective *wt* and *mts***.**
